# Evaluations of Spatial Accessibility and Equity of Multi-Tiered Medical System: A Case Study of Shenzhen, China

**DOI:** 10.3390/ijerph19053017

**Published:** 2022-03-04

**Authors:** Meng Tian, Lei Yuan, Renzhong Guo, Yongsheng Wu, Xiaojian Liu

**Affiliations:** 1 Lab for Optimizing Design of Built Environment, School of Architecture and Urban Planning, Shenzhen University, Shenzhen 518060, China; meng.tian.c@hotmail.com; 2 Research Institute for Smart Cities, School of Architecture and Urban Planning, Shenzhen University, Shenzhen 518060, China; guorz@szu.edu.cn; 3 Guangdong Key Laboratory of Urban Informatics, Shenzhen University, Shenzhen 518060, China; 4 MNR Technology Innovation Centre of Territorial & Spatial Big Data, Shenzhen University, Shenzhen 518060, China; 5 Shenzhen Centre for Disease Control and Prevention, Shenzhen 518055, China; cdc@szcdc.net (Y.W.); xjliu@szcdc.net (X.L.)

**Keywords:** spatial accessibility, medical system, multi-tiered two-step floating catchment area (MT2SFCA) method, equity, healthcare

## Abstract

The Chinese government has implemented a medical system reform to improve the equity of healthcare resources since 2009. We selected Shenzhen as our study area and evaluated the accessibility and equity of the multi-tiered medical system in China using a novel multi-tiered two-step floating catchment area (MT2SFCA) method. We proposed the benchmark and applied the independent variables of travel time and facility attractiveness, along with a combination of the two factors, as tolerances to determine the new logistic cumulative distribution decay functions. Community health centers (CHCs) and hospitals were included while integrating their features. Results revealed that the MT2SFCA method was able to determine the particular advantages of CHCs and hospitals in the multi-tiered medical system. The CHCs offset the lower accessibility of hospitals in suburban areas and hospitals balanced the regional inequity caused by the CHC. Travel time is the main consideration of patients who have access to CHCs, whereas facility features are the main considerations of patients who have access to hospitals. Notably, both CHCs and hospitals are crucial for the whole multi-tiered medical system. Finally, we suggested modifications in different travel modes, weights of contributing factors, and the validation of decay functions to improve the MT2SFCA method.

## 1. Introduction

### 1.1. Spatial Equity and Accessibility to Health Services

In terms of accessing public facilities, spatial equity may be defined as the degree to which facilities are distributed in an equal way, corresponding to the requirements of facilities over different areas. It is a critical problem that must be considered by urban planners and policymakers to determine the under-provisioned areas and then, make decisions to allocate the public services more effectively [1]. People experiencing easily-accessed and adequately-resourced facilities are generally reported to have higher happiness levels and better health status [2,3,4]. Achieving equity in healthcare services is one of the fundamental goals of Universal Health Coverage (UHC) [5]. The equity of access to healthcare facilities has become one of the most crucial objectives of healthcare systems in many countries, with the aim of achieving social equity by promoting the livelihood and health conditions of people. In the development targets of the 14th Five-Year Plan, the Chinese government mentioned the equity of healthcare facilities, while highlighting the means of de-centralization of healthcare facilities in city centers [6].

Being the most popular factor used for evaluating the spatial equity of public service facilities, accessibility reflects the demand–supply relations and spatial distributions of facilities along road networks [7,8]. The 2SFCA method has become one of the most popular methods to analyze the accessibility of various infrastructures. The basic model of the 2SFCA method [9] originated from the dichotomous transformation of the gravity model [10]. Subsequently, many advanced versions have been proposed to improve the basic model. Luo and Qi [11] firstly proposed using several travel time zones with various weights to denote the distance decay effects. Continuous gradual decay functions were exploited in Gaussian-based 2SFCA (G2SFCA) [12] and Kernel density-based 2SFCA (KD2SFCA) [13] methods. Wan et al. [14] proposed the three-step-floating catchment area (3SFCA) method, which further explains the supply–demand relation by considering the competitive effects from nearby facilities. Delamater [15] indicated that all the traditional 2SFCA and 3SFCA methods have the shortcoming of container-like systems; therefore, he introduced the modified 2SFCA (M2SFCA) method, while considering the suboptimal conditions of healthcare distributions. Luo and Whippo [16] applied dynamic catchment sizes to balance the discrepant allocations of healthcare facilities in urban and rural areas. Bauer and Groneberg [17] integrated the M2SFCA method with a logistic-based decay function that replaced the impedance coefficients (using available distribution parameter) so that the catchment sizes would vary independently, based on the distributions of facilities within the catchment areas. 

Except travel distance, accessibility is also suggested to consider other effects, such as medical management, service quality, consumer income, and cultural factors. Penchansky and Thomas [18] proposed a comprehensive definition of ‘access to healthcare’ that described the fit between the patient and the healthcare system in a series of aspects, including availability, accessibility, accommodation, affordability, and acceptability. Recently, some studies have considered the characteristics of healthcare when evaluating the spatial accessibility and equity of a health system to obtain results that are much closer to the realistic situation. Luo [19] innovatively applied the Huff model to the 3SFCA method to describe the two factors that affect the decisions of patients to select healthcare facilities, which are the travel distance and the attractiveness of facility. Shin and Lee [20] utilized the number of patient visits normalized by hospital capacity to substitute the travel time in the distance decay function, to calculate the accessibility of emergency medical services in South Korea. 

Because the medical system is one of the most important livelihood projects of a country, during the urbanization in China, scholars have paid significant attention to the equity and accessibility of healthcare facilities. Rong et al. [21] evaluated the accessibility of comprehensive hospitals in Zhengzhou, Henan Province, China, and observed that the medical facilities demonstrated a concentric structure in the central area of the city. Lu et al. [22] compared the accessibility before and after the referral reform of the hierarchical health system of China, and deduced that the referral reform significantly improved the accessibility of health services. Wang et al. [23] investigated the accessibility of community/township health centers in Sichuan Province and revealed that the spatial disparity was associated with population density and ethnic minority. Chen et al. [24] evaluated the accessibility of secondary and tertiary hospitals in Shenzhen, based on the big data of taxi trajectory. Similar to all other cities in China, the accessibility of hospitals in Shenzhen also illustrated uneven distributions, and notably, the central area has better access to healthcare services. 

### 1.2. Research Aims and Objectives

According to the literature, we were able to deduce that most studies only concentrated on the accessibility of hospitals; these studies overlooked the benefits of primary healthcare facilities such as community health stations and clinics. Moreover, in most cities in China, the multi-tiered medical system only includes hospitals, among which the primary hospitals are responsible for providing basic, preventive, and rehabilitation healthcare services to the communities; however, there are only a few primary hospitals. For example, in the research focusing on the equity of healthcare resources in Beijing, Lu et al. [22] found that the patients in two remote towns cannot reach any primary hospital within 30 min. Because Shenzhen is a special zone and owing to the early and relatively successful implementation of health system reform in the city, the multi-tiered medical system in Shenzhen has more advantages compared to other cities. It now incorporates three-tier hospitals as well as hundreds of community health centers (CHCs). 

The aim of our study is to provide a reliable and viable method to analyze spatial data to reveal possible inequity and inaccessibility of healthcare facilities in Shenzhen, based on the numbers of real outpatient visits to healthcare facilities. In this study, we investigated the accessibility and equity of the multi-tiered medical system in China after the health system reform was implemented. First, we explained the multi-tiered medical system in Shenzhen, China, including the system structure and relevant policies. Then, we introduced the multi-tiered two-step floating catchment area (MT2SFCA) method to determine the accessibility and equity of the multi-tiered medical system. We proposed the independent variables of travel time and facilities attractiveness, along with a combination of the two factors as the benchmark, Scenario 1 and Scenario 2 of tolerance to determine the new logistic cumulative distribution decay function. Finally, we discussed the reasons that affected people’s selection of healthcare facilities by comparing the two scenarios used with the benchmark in MT2SFCA model. The results can then be used by policy makers and scholars to address the limitations in accessible healthcare and thus, promote the equity and accessibility of the multi-tiered medical system.

## 2. Multi-Tiered Medical System in China

To achieve the global goals of UHC, the strategy of Healthy China 2020 was proposed, with the aim of providing basic healthcare coverage to all residents living in urban and rural areas by the year 2020. A new round health system reform was launched in 2009 [25]. The objectives of this reform included improving the accessibility and equity of healthcare services, providing well-rounded health services, and improving the medical insurance system [26]. 

The healthcare system in China is a multi-tiered medical system having a two-way patient referral mechanism, in which the healthcare facilities are categorized into three tiers. The first-tier includes primary hospitals, CHCs, and clinics to provide healthcare services for common diseases, disease prevention, and rehabilitation. In addition, CHCs also provide services, such as vaccination, health promotion, family planning, medical education, family doctor, home healthcare, and chronic diseases detection and treatment. The second-tier and third-tier hospitals (also known as secondary hospitals and tertiary hospitals) are regional/district hospitals and municipal hospitals, both of which provide specialized and high-level healthcare services. In addition to these services, the tertiary hospitals offer medical research and education. 

The two-way patient referral mechanism aims to optimize the allocation of healthcare resources. Patients are freely but encouraged to seek health services in primary healthcare institutions, and then, they are referred to secondary and tertiary hospitals if needed. Other than the referrals that may be generated from primary to secondary and tertiary healthcare facilities, they can also be referred in the reverse direction, that is, the hospitals in higher tiers can generate referrals to CHCs for subsequent treatments such as rehabilitation and chronic diseases management at an affordable cost. 

Shenzhen has experienced rapid urbanization for decades and has become one of the most populated Tier 1 cities in China. Therefore, Shenzhen is ideal as a case study for the analysis of urban problems in developing countries [27]. Because it is a new and high-density city with rapid population growth, allocating healthcare resources properly is a tough challenge in Shenzhen. Shenzhen government has moved forward on a healthcare reform program by implementing various actions, including financial and policy supports, and made some achievements. In 2018, the densities of hospital beds, physicians, nurses, and health professionals achieved 3.65, 2.79, 3.09, and 8.82 per 1000 of the population, respectively, for long-term residents in Shenzhen [28]; more than 95% families can reach the nearest healthcare facilities within 15 min [29]. 

**Figure 1:**
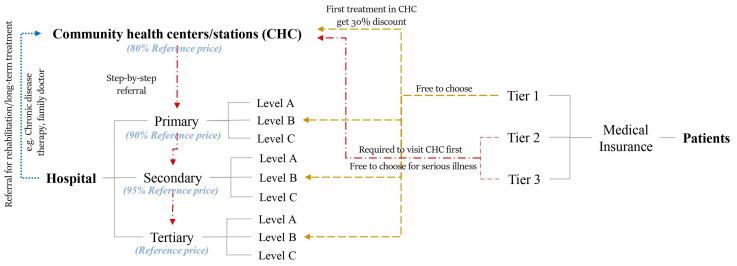
Introduction of multi-tiered medical system in Shenzhen, China.

The process of the outpatient service of the multi-tiered medical system in Shenzhen is shown in Figure 1. The system consists of community health centers (CHCs) and hospitals. Hospitals are ranked into three categories, which are primary, secondary, and tertiary hospitals, according to their capacities and service qualities. Each category is subdivided into A, B, and C levels with respect to its medical service level. The outpatient services of local residents depend on the types of their medical insurances. Three ranks of medical insurance can be selected for the residents in Shenzhen. The Tier 1 insurance is the best, followed by Tier 2 and Tier 3. Better insurance also entails more monthly fees and larger proportions of reimbursements while in use. During the outpatient services, the patients with the Tier 1 insurance are free to choose any medical services including CHCs and hospitals. To encourage people to visit CHCs, the government implemented a policy that 30% of the patient’s expenses would be covered by the local government when they visit CHC for the first-time outpatient service. The patients with Tier 2 and Tier 3 medical insurances are first asked to visit CHCs for outpatient service and then, the step-by-step referral process (from lower to higher ranked facilities) is followed if needed. In addition, they are still free to choose healthcare facilities for serious illnesses. If patients need subsequent or long-term rehabilitation, they may be transferred to the lower-level facilities. Except for the policies implemented for the patients, some policies are put forward to improve the competitiveness of the lower-tier facilities. The prices of medicines and therapies in tertiary hospitals follow the referenced price-level of the province. The prices of secondary and primary hospitals are set as 95 and 90% of the reference prices, respectively. Notably, the prices of CHCs are set as low as 80% of the reference prices. As stated before, after the Shenzhen government provided sufficient support for primary-care infrastructure construction, personnel training, financial support, and policy implementation, the acceptance of CHCs by local residents has grown. In 2018, about 40% of first-time outpatient services were completed by CHCs for the whole city [30].

## 3. Methods

### 3.1. Study Area and Data Sources

**Figure 2:**
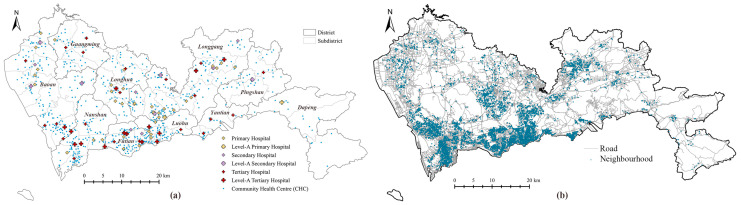
Introduction of research area and objects: (**a**) locations of healthcare facilities in Shenzhen; (**b**) locations of neighborhoods in Shenzhen.

Shenzhen (22°32′ N, 114°30′ E) is located on the central coast of the southern Guangdong Province. As a special economic zone with fast-growing economy, it has become one of the biggest cities in China. The long-term residents have exceeded 12 million [31]. Shenzhen comprises of 10 districts and 74 subdistricts (Figure 2a) that cover an area of ~2000 km^2^. Three districts, namely, Luohu, Futian, and Nanshan, are considered as the central areas of the city.

The multi-tiered medical system in Shenzhen consists of hospitals and CHCs. In this study, all 84 general hospitals (including 6 Chinese general hospitals) and 692 CHCs were applied to investigate the accessibility of the multi-tiered medical system through the MT2SFCA method. The numbers of outpatient visits and physicians for each hospital and CHC were obtained from the Statistics Bureau of the Shenzhen Municipality and the 2018 Annual Health Statistics of Shenzhen Municipality [28]. The locations of healthcare facilities were extracted from Open Maps [32] using geocoding (Figure 2a). The catchment areas of hospitals and CHCs were 30 min of driving and 30 min of walking, respectively [33]. According to the Shenzhen Traffic Performance Index System [34], the driving speeds were determined by the average value in peak hours, which were 75 km/h for highways, 45 km/h for arterial roads, 30 km/h for city roads, and 20 km/h for local streets. The walking speed was set as 5 km/h. The road network was obtained from OpenStreetMap [35]. Information about 6328 neighborhoods (Figure 2b) was obtained from the public data of the most popular real estate website [36] in China. The population was estimated using the average values of 2017 and 2018, as reported by the Shenzhen Centre for Disease Control and Prevention. The accessibility was calculated using ArcGIS 10.7 [37]. 

### 3.2. Calculating Accessibility of Multi-Tiered Medical System

#### 3.2.1. Attractiveness of Health Facilities

The designed capacity of a hospital depends on the potential demand of surrounding neighborhoods, which is reflected by the final size of the hospital, including the numbers of beds, physicians, nurses, and health professionals. Generally, these numbers have strong correlations with each other. For the general hospitals in Shenzhen, the coefficients of determination (R^2^) of the linear relationships between the numbers of physicians and nurses, numbers of physicians and health professionals, and numbers of physicians and beds were 0.97, 0.99, and 0.80, respectively. The reason of the lower R^2^ of the correlations between the numbers of physicians and beds is that the number of beds is also determined by the specialties of the hospitals. Therefore, the number of physicians represents the designed capacity of the hospital more accurately. Notably, the real demand of the hospital is represented by the annual actual outpatient visits to the hospital. The attractiveness of the hospital can be expressed by Equation (1), which is determined by the ratio of the number of actual visits to the number of physicians in the hospital.

(1) $$q_{ij}=\frac{V_{j}}{S_{j}}\;\;\;\;$$

where j is the healthcare location, i is the population location, $q_{ij}$ is the attractiveness of health facility, $V_{j}$ is the annual actual outpatient visits of the facility, and $S_{j}$ is the number of physicians of the facility.

By being divided by the number of physicians, the number of visits was normalized by the hospital capacity, so that the attractiveness due to the hospital itself could be evaluated to portray the high quality of services and expert skills of health professionals. The definitions of attractiveness were discussed in detail under two scenarios in Section 3.2.2.

#### 3.2.2. Tolerance Decay Function

A tolerance decay function $f_{i}\left(w_{ij}\right)$ is proposed as a substitute for the distance decay function applied in the 2SFCA method to consider both the attractiveness of the healthcare facility and the distance between the supply and demand points. Therefore, before any other analysis, we first determined the tolerance. 

In this study, we analyzed and compared two scenarios of tolerance, as follows:

**Scenario** **1.** *Tolerance was regarded as being affected by the attractiveness* $\left(q_{ij}\right)$ *of the health facility including facility features and distance.*

In this scenario, we assumed that the actual outpatient visits of health facilities were affected by the distance and service quality [20]. Thus, the attractiveness ($q_{ij})$ included the effects by both the facility itself and its distance. The tolerance of residents who visited the healthcare facilities was calculated by Equation (2).

(2) $$w_{1ij}=Norm\left(-q_{ij}\right)\;\;\;\;$$

where $w_{1ij}$ is the tolerance of residents who visited the healthcare facilities under the conditions of Scenario 1, and $Norm\left(-q_{ij}\right)$ is the normalization of $-q_{ij}$.

**Scenario** **2.** *The attractiveness* $\left(q_{ij}\right)$ *of the health facility was considered to only represent the effects of the facility features, such as service quality, physician level, and crowdedness.*

The tolerance of the visitors was considered as the comprehensive assessment of residents visiting a healthcare facility, which consisted of two factors, the distance between the population and healthcare facility and the attractiveness of the healthcare facility. There are several ways to conduct a comprehensive assessment of a criteria affected by several factors, and multiplying or adding weighted factors are the most common methods [38,39]. Compared to multiplication weighted factor, addition weighted factors can obtain a dataset having lower dispersion and smaller fluctuations. Therefore, the addition formula was chosen to determine the tolerance of the patients visiting a healthcare facility (Equation (3)). Notably, we assumed that the attractiveness of the facility and the distance of the facility from the residents’ locations had equal influences on the tolerance.

(3) $$w_{2ij}=Norm\left(t_{ij}\right)+Norm\left(-q_{ij}\right)\;\;$$

where $w_{2ij}$ is the tolerance of residents visiting health facilities in Scenario 2, $t_{ij}$ is the travel time from the population location to the supply service, $Norm\left(t_{ij}\right)$ and $Norm\left(-q_{ij}\right)$ are the normalizations of the datasets $t_{ij}$ and $-q_{ij}$. They were normalized to [0,1] to eliminate the influence of their numerical values.

The tolerance decay function $f_{i}\left(w_{ij}\right)$ was determined by the logistic cumulative distribution function (CDF) [Equation (4)], which is a downward sigmoid function (S-shape) based on a logistic distribution method [17]. Because the CDF used the median and standard deviation (SD) of the tolerance dataset $\left(w_{ij}\right)$, a uniquely shaped decay function, $f_{i}\left(w_{ij}\right)$, was obtained for every population location i. 

**Figure 3:**
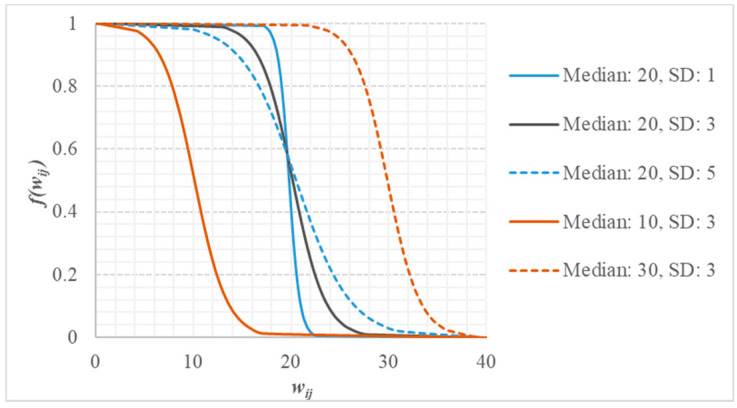
Effects of median and standard deviation (SD) on tolerance decay function; x axis represents the tolerance $\left(w_{ij}\right)$ and y axis represents tolerance decay function $f_{i}\left(w_{ij}\right)$.

Figure 3 illustrates an example of the changes in $f_{i}\left(w_{ij}\right)$ with various medians and SDs of $w_{ij}$. We observed that the median shifted the function in horizontal direction, and the SD changed the steepness. For a fixed SD, a larger median value resulted in stronger tolerance for longer distances and lower attractiveness. For a fixed median, a smaller SD resulted in a steeper curve, indicating that the residents were more sensitive to the tolerance factors.

(4) $$f_{i}\left(w_{ij}\right)=\frac{1+\exp\left(-\frac{\mu \pi }{\sigma \sqrt{3}}\right)}{1+\exp\left(\frac{\left(w_{ij}-\mu \right)\pi }{\sigma \sqrt{3}}\right)}$$

where μ is the median of $w_{ij}$, and σ is the SD of $w_{ij}$.

#### 3.2.3. Accessibility

The accessibility of healthcare facilities was determined based on the M2SFCA model [15], which considers the supply–demand relations, along with suboptimal configurations, as shown in Equation (5). In this accessibility equation, the traditional distance decay function was substituted by the tolerance decay function, which considered the attractiveness and distance of the health facilities. As discussed in Section 3.2.2, the tolerance decay function, $f_{i}\left(w_{ij}\right)$, varied for each catchment area. The suboptimal configuration was evaluated by $f_{G}\left(w_{ij}\right)$, which represented the general benchmark tolerance of patients who visited the healthcare facilities, and it was calculated using all the tolerance values calculated for the entire study area regardless of catchment area [Equation (6)].

(5) $$A_{i}=\sum_{j\in \left\{t_{ij}\leq t_{0}\right\}}\frac{S_{j}f_{i}\left(w_{ij}\right)f_{G}\left(w_{ij}\right)}{\sum_{i\in \left\{t_{ij}\leq t_{0}\right\}}P_{i}f_{i}\left(w_{ij}\right)}$$

where $S_{j}$ is the number of physicians of the facility, $P_{i}$ is the population of the demand location, $f_{G}\left(w_{ij}\right)$ is the global tolerance decay function, $t_{ij}$ is the travel time between the residents and the healthcare locations, $t_{0}$ is the maximum travel time of the catchment area, and $A_{i}$ is the accessibility of the demand location.

(6) $$f_{G}\left(w_{ij}\right)=\frac{1+\exp\left(-\frac{\mu_{G}\pi }{\sigma_{G}\sqrt{3}}\right)}{1+\exp\left(\frac{\left(w_{ij}-\mu_{G}\right)\pi }{\sigma_{G}\sqrt{3}}\right)}$$

where $\mu_{G}$ and $\sigma_{G}$ are the medians and SDs of all the $w_{ij}$ values calculated for the entire study area regardless of catchment area; it is a constant value.

#### 3.2.4. Accessibility of Multi-Tiered Health System

**Figure 4:**
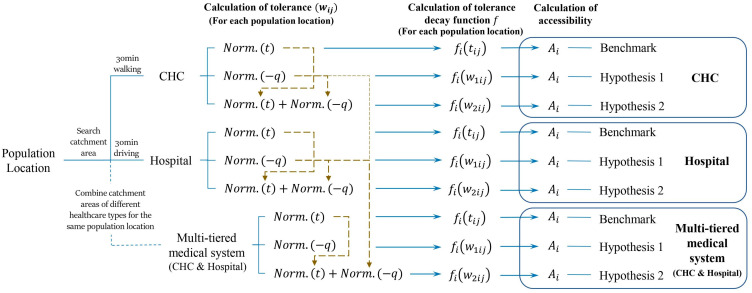
Flowchart explaining the process of calculating the accessibility of CHC, hospital, and the multi-tiered medical system.

The multi-tiered health system consists of two main types of healthcare facilities, i.e., hospitals and CHCs. To calculate the accessibility of the multi-tiered health system, we proposed the MT2SFCA method to adapt to the system. The process of evaluating accessibility is demonstrated in Figure 4. First, the respective accessibilities of CHCs and hospitals were calculated separately, and then, the results were compared to the accessibility of the multi-tiered health system (CHCs and hospitals).

For each of the three healthcare configurations, the accessibilities are determined in three ways by varying the tolerance decay function $f_{i}\left(w_{ij}\right)$. First, we determined the benchmark value of accessibility by using the travel time, $t_{ij}$, as the tolerance in Equation (5), which is similar to the traditional 2SFCA method. Then, another two methods consider the attractiveness and travel time of healthcare as tolerance as indicated by Equations (2) and (3) of Scenarios 1 and 2. It is important to note that the catchment areas of CHC and hospital were different, which are determined by ‘30 min by walking’ and ‘30 min by driving’. When we calculated the accessibility of the multi-tiered medical system, the catchment areas of both the CHCs and hospitals were summed up for each neighborhood, referring to the 2SFCA method with various catchment areas [14]. 

In terms of the multi-tiered medical system, the normalization of the parameters in tolerance should be carried out after aggregating all the CHCs and hospitals together within their catchment areas for each neighborhood. For Scenario 1, the tolerance was calculated by the normalization of $-q_{ij}$ for the combined dataset of the CHCs and hospitals, because $q_{ij}$ represented the attractiveness of the facility, while considering all the influencing factors. For Scenario 2, the minus attractiveness, $-q_{ij}$, of healthcare facilities within each catchment area was normalized for CHCs and hospitals respectively, and the travel time, $t_{ij}$, was normalized for the combined dataset of the CHCs and hospitals. Then, the tolerance was calculated by the sum of the normalized $-q_{ij}$ and normalized $t_{ij}$ as indicated by the yellow dashed line in Figure 4. It is important to mention that the combined dataset of CHCs and hospitals was used when the $t_{ij}$ was normalized because travel time is one of the main reasons for patients to prefer a healthcare facility, which is the core competitiveness of CHCs. However, the respective datasets of CHCs and hospitals were used for the normalization of $-q_{ij}$, because the attractiveness of the healthcare facilities in Scenario 2 mainly represented the characteristics of the healthcare facilities themselves. The CHCs and hospitals have their own specialties and drawbacks. CHCs mainly deal with common diseases and hospitals are skilled in treating specialized diseases. Generally, people spend lesser time in CHCs than hospitals. Thus, it is not suitable to normalize the attractiveness of CHC and hospital together. 

#### 3.2.5. Gini Index of Accessibility

Gini index is one of the most common measurements to evaluate social inequity [40]. In economics, Lorentz curves are plotted to obtain the Gini index, which presents the cumulative percentage of total wealth against the cumulative of population (possessing the wealth), beginning with the poorest recipient [41]. The equality line represents the situation that each recipient has equal wealth. Gini index is calculated by the area between Lorentz curve and the line of absolute equality, being divided by the triangle area under the equality line, which is independent of the measure unit. The Gini index value of 0 refers to the perfect equality circumstances, in which the Lorentz curve and the straight line of absolute equality are coincident. More unequal conditions are indicated by higher Gini index.

In recent years, Gini index has also been applied to evaluate the spatial equity of health services. In this study, Gini index was used to evaluate the spatial equity of healthcare facilities at the subdistrict level. The Gini index was calculated by the variables of accessibility. The Lorentz curve was plotted using the cumulative percentage of accessibility against the fraction of total population at the subdistrict level, which was arranged in ascending order of accessibility. The Gini index was calculated using RStudio [42].

## 4. Results

**Figure 5:**
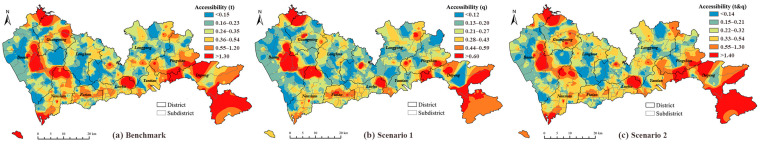
Accessibility of CHCs: (**a**) benchmark of accessibility; (**b**) accessibility calculated for Scenario 1; (**c**) accessibility calculated for Scenario 2.

**Figure 6:**
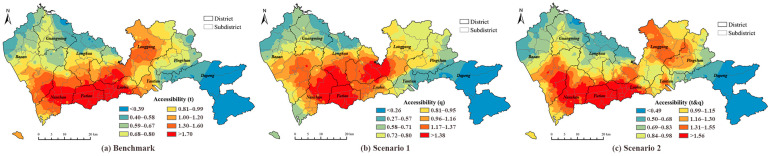
Accessibility of hospitals: (**a**) benchmark of accessibility; (**b**) accessibility calculated for Scenario 1; (**c**) accessibility calculated for Scenario 2.

**Figure 7:**
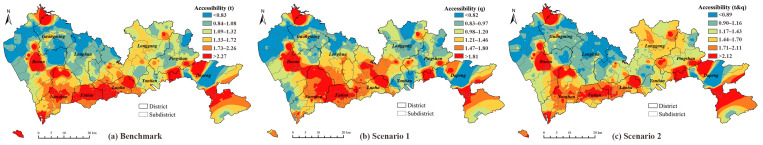
Accessibility of multi-tiered medical system: (**a**) benchmark of accessibility; (**b**) accessibility calculated for Scenario 1; (**c**) accessibility calculated for Scenario 2.

For the ~11.7 million long-term residents living in 6328 neighborhoods in Shenzhen, the average number of physicians per 1000 people were 2.76 and 0.38 for hospitals and CHCs, respectively. The results of accessibility calculated using the MT2SFCA method are illustrated in Figure 5, Figure 6 and Figure 7 for the CHCs, hospitals, and the entire multi-tiered medical system (including both CHC and hospital), respectively. In each figure, the subpart (a) represents the benchmark value of accessibility. The subparts (b) and (c) of accessibility were obtained for the Scenarios 1 ($w_{1ij}$) and 2 ($w_{2ij}$), respectively, using the MT2SFCA method proposed in this study. 

In terms of CHCs, the accessibility determined for Scenario 1 was obviously smaller than the benchmark value, and the results of Scenario 2 were close to the benchmark, based on the values of quantiles (Figure 5). In these three methods, the spatial distributions of the values in sextiles were similar, and only smaller differences could be found. For example, in Scenario 1, the areas of the highest and the lowest quantiles were smaller compared to benchmark, e.g., in Longhua, Longgang, and Nanshan districts. The results showed that the attractiveness and travel time affected the willingness of residents to visit the CHCs in the same way, which implied that the main reason for people choosing CHCs was travel time for both scenarios. 

The accessibility of the hospitals calculated using three methods illustrated the apparent discrepancies. The values of the quantiles of the benchmark were higher than those obtained for Scenario 1, and similar for Scenario 2. The spatial distributions of the accessibility values were also different for the three methods. For the benchmark result shown in Figure 6a, the healthcare facilities in the central areas of Shenzhen had better accessibility, that is, the districts of Nanshan, Futian, and Luohu, where the hospitals were densely located. Notably, the distribution of higher accessibility followed the distribution of tertiary hospitals. For the result of Scenario 1 [Figure 6b], the highest quantile of accessibility expanded from the city center to the Longhua and Longgang districts. Compared to the benchmark, the distribution of higher accessibility in Scenario 1 was similar to the distributions of tertiary and primary hospitals. The distribution pattern of accessibility obtained by Scenario 2 indicated similar results to the benchmark. It also implied that in Scenario 1, the attractiveness of the hospitals due to other factors, such as hospital level and service quality, was more important than the travel time when the residents preferred a hospital, whereas in Scenario 2, the travel time indicated a more significant effect compared to the attractiveness of the healthcare facility. 

The accessibility of the multi-tiered medical system is demonstrated in Figure 7. The results combined the respective results of the CHCs and hospitals given in previous sections. The higher accessibilities were found not only in the central areas of the city in the Nanshan, Futian, and Luohu districts as the results of hospitals, but also in suburban areas, such as Baoan, Pingshan, and Dapeng districts, similar to the results of CHCs. In terms of the quantile values and spatial distributions, compared to Scenario 1, the result of Scenario 2 was closer to the result of benchmark. 

**Figure 8:**
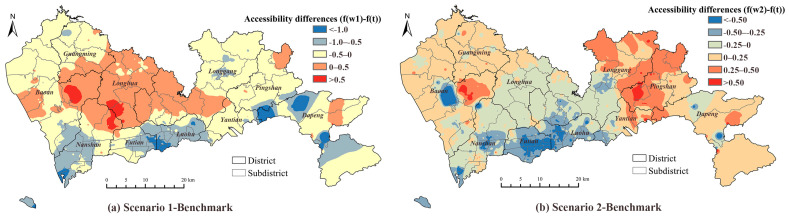
Accessibility differences between: (**a**) Scenario 1 and the benchmark; (**b**) Scenario 2 and the benchmark.

Figure 8 demonstrates how the results of Scenarios 1 and 2 deviated from that of the benchmark. The positive and negative deviations indicated obvious spatial clusters. For Scenario 1, in which we assumed that the healthcare attractiveness, including both travel time and facility features, was calculated by normalized outpatient visits; the accessibility declined mainly in the central areas of Shenzhen (Nanshan, Futian, and Luohu districts). The increments of accessibility were located in suburban areas (Longhua, part of Longgang, and part of Baoan districts). The negative deviations of accessibility occupied more than half the area of the whole city. In terms of Scenario 2, the accessibility decreased mainly in the city center, similar to Scenario 1 and also in some suburban areas, such as the Longhua and Longgang districts. The positive deviations were found in the western and eastern suburban areas. 

**Figure 9:**
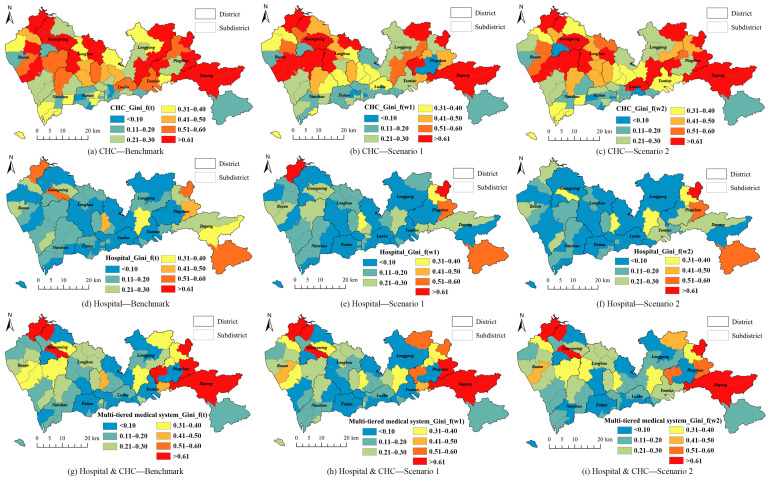
Gini indexes of CHCs, hospitals, and the entire multi-tiered medical system for subdistrict in Shenzhen, China: (**a**) Benchmark of Gini index for CHC; (**b**) Gini index of CHC calculated for Scenario 1; (**c**) Gini index of CHC calculated for Scenario 2; (**d**) Benchmark of Gini index for hospital; (**e**) Gini index of hospital calculated for Scenario 1; (**f**) Gini index of hospital calculated for Scenario 2; (**g**) Benchmark of Gini index for multi-tiered medical system; (**h**) Gini index of multi-tiered medical system calculated for Scenario 1; (**i**) Gini index of multi-tiered medical system calculated for Scenario 2.

The Gini indexes of subdistricts in Shenzhen were obtained based on the accessibility of the CHCs, hospitals, and the entire multi-tiered medical system as demonstrated in Figure 9. In general, the accessibility of the CHCs indicated obvious disparities, compared with the accessibility of hospitals, especially in northern areas. The Gini indexes of most subdistricts were 0.4 or higher. Owing to the larger service area, the residents living far from the city center were able to reach the hospitals, and thus, their equity of hospital accessibility provided better results. By considering both the CHCs and hospitals in the multi-tiered medical system, we were able to deduce that the number of the subdistricts that offered unequal health services was small. Only several remote regions indicated an equity problem. Compared to the accessibility distributions, we were able to deduce that the regions having higher accessibility (city center) generally experienced better equity. However, the more homogeneous distributions of accessibility resulted in lower values of the Gini index, although the values of accessibility may not be high for the hospital accessibility in northern areas, i.e., the Longhua and Longgang districts. The subdistricts having both low and high values of accessibility indicated low equity, e.g., the subdistricts in the Dapeng district.

**Figure 10:**
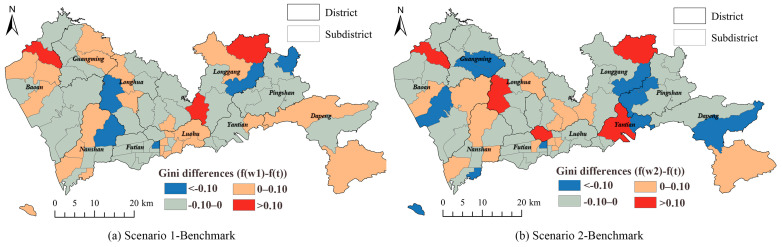
Differences between Gini indexes of: (**a**) Scenario 1 and benchmark; (**b**) Scenario 2 and benchmark of the multi-tiered medical system.

The discrepancies in the Gini indexes calculated for the benchmark, Scenario 1, and Scenario 2 are portrayed in Figure 10. Different from accessibility, the discrepancies did not show obvious spatial aggregations. The results of both the scenarios in most subdistricts were lower than the benchmark results. The results of Scenario 2 indicated more subdistricts having discrepancies higher than 0.1 or lower than −0.1, compared to Scenario 1. The results implied that travel time was not the only factor that affected the people’s decisions to visit a healthcare facility. Factors, such as facility features and financial conditions of patients, affected the competitiveness of the facilities. Thus, the equity of the healthcare accessibilities of Scenarios 1 and 2 offered better results than the benchmark, which only considered travel time as a major influencing factor.

## 5. Discussion

**Figure 11:**
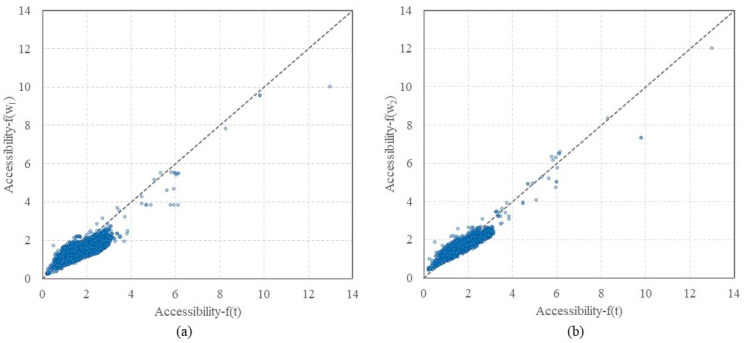
Comparisons of benchmark with (**a**) Scenario 1 and (**b**) Scenario 2, plotted along with a dashed 1:1 line for reference.

By considering the time and attractiveness, along with a combination of the two factors, the MT2SFCA method described the supply–demand relations of medical resources in multiple aspects. The results distinguished the main factor that affects the decisions of patients to select different healthcare facilities. The accessibilities of the multi-tiered medical system calculated under the Scenarios 1 and 2 are plotted against the benchmark value in Figure 11. Similar to the spatial distribution, more than half the results were smaller than the benchmark values in Scenario 1. In Scenario 2, the points were above or underneath the 1:1 line, which indicated that travel time played a more predominant role in Scenario 2, compared to Scenario 1. 

**Figure 12:**
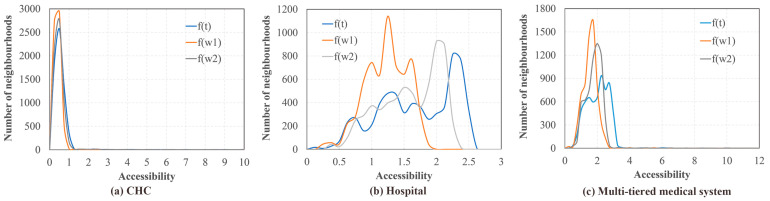
Density plots of MTSFCA results for (**a**) CHCs, (**b**) hospitals, and (**c**) the multi-tiered medical system in Shenzhen, China.

The density plots of the MT2SFCA results are illustrated in Figure 12. The results of CHCs, hospitals, and the entire multi-tiered medical system presented unique distributions. The accessibility indicated similar shapes for the benchmark $f\left(t\right)$, Scenario 1 $f\left(w_{1}\right)$, and Scenario 2 $f\left(w_{1}\right),$ in terms of the CHCs. Scenario 1 portrayed the largest peak value, followed by Scenario 2. Due to the lower number of physicians in the CHCs (compared to the hospitals), the accessibility of CHCs was aggregated between 0 to 1. The density plot of the hospital accessibility presented different distributions in Scenario 1 compared to those for the benchmark and Scenario 2. The accessibility of Scenario 1 was mostly clustered between 1 and 1.5, whereas it was mainly distributed around 2 and 2.5 for Scenario 2 and the benchmark, respectively. The accessibility of the entire multi-tiered medical system was the combination of the individual results of the CHCs and hospitals. Notably, the peak values of the neighborhood numbers were lower than those of CHCs, but higher than those of the hospitals. The results of Scenario 2 were consistent at the middle level, among all the results of benchmark, Scenario 1, and Scenario 2 for each healthcare configuration. 

The results also highlighted the importance of primary healthcare facilities including CHCs and primary hospitals. With the increment of referral rate, they played critical roles in improving the accessibility and equity of the multi-tiered medical system, especially for the suburban area. Similar results were also found in the study of Beijing [22]. Thus, increasing the number of primary healthcare facilities was a main solution to solve the inequity of medical services in China [23,43].

For the benchmark, Scenario 1, and Scenario 2, the distance between healthcare facilities and patients was a critical factor that cannot be ignored when patients choose facilities, especially for CHCs. A study focusing on the primary healthcare in Sweden also found that distance is the most important factor in choosing a primary healthcare provider [44]. The distance effect also applicable to online therapy. By investigating a third-party online healthcare platform in China, Chen et al. found that the distance between doctors and patients is negatively associated with online service utilization [45]. In addition, distance has become more important due to the lockdown restrictions during COVID-19. Palm et al. found that many residents in Toronto and Vancouver defer medical care because the healthcare and pharmacy services are too far to reach by walking after stopping riding public transit during COVID-19 [46].

Although the travel distance is important, results revealed that the facility features were considered more when patients were going to choose hospitals. In research in Italy, it was found that many patients are willing to travel far and wait longer for seeking better healthcare services [47]. The better services hospitals offer, the more likely patients will choose [48]. This circumstance also occurs not only in intra-city healthcare, but in cross-city healthcare in China [49]. 

The accessibility and equity of the multi-tiered medical system consisting of CHCs and hospitals in Shenzhen were investigated in this study. Several advantages of the MT2SFCA method were highlighted. First, the MT2SFCA method was developed based on the M2SFCA method, which settled the limitation of traditional E2SFCA and 3SFCA methods by evaluating the overall effectiveness caused by any system change, i.e., increasing or removing any supply location [15]. Second, the uniquely shaped tolerance decay function for each catchment area solved the distinct allocations of healthcare resources. Other than the multiple attempts of applying various impedance coefficient when using Gaussian function and inverse function as the distance decay function, the logistic cumulative distribution function used in this study adjusted the curve shape by the distribution of dataset values. Moreover, it considered the specific distributions of the travel time or attractiveness of the CHCs and hospitals when evaluating the accessibility of the multi-tiered medical system. In terms of the benchmark, the greater the median travel time to the facilities within the catchment area, the more likely the patients were to travel further. If the healthcare facility provided spatial aggregations, which resulted in smaller standard deviations, the patients preferred to spend less time traveling to the healthcare facility. The tolerance decay of the people who visited the healthcare facility in Scenarios 1 and 2 worked on the same principle to the tolerance decay for the benchmark, whereas the measurements were replaced by the facility attractiveness and travel time. Finally, as an improvement in the M2SFCA method, we replaced one of the decay functions $f_{i}\left(w_{ij}\right)$ with the global decay function $f_{G}\left(w_{ij}\right)$ that represented the tolerance level of the whole research area instead of only the catchment area. The global decay function balanced the effects of the peak values for some catchment areas, because it was a fixed curve of all catchment areas [17]. In this study, the selection of the global tolerance function was based on the entire area of Shenzhen. 

## 6. Limitations of the Study

The limitations of this study must be mentioned as well. Although this study did not consider the variable travel time for urban and suburban areas, this simplification was still explainable for Shenzhen. First, the 2000 km^2^ area was not big for a first-tier city, compared to the area of 16,410 km^2^ of Beijing, 7430 km^2^ of Guangzhou, and 6340 km^2^ of Shanghai. Most areas in Shenzhen were accessible via a one-hour drive. Second, the CHCs in Shenzhen were accessible for most neighborhoods. Finally, the variable tolerance decay curves determined by the CDFs within the same travel time were calculated while considering the unequal distributions of healthcare facilities in the city. Thus, it is not necessary to intentionally include that the people living in rural area may be go further for visiting healthcare. However, variations in the travel time are still suggested to be considered in further studies or for other cities. The benefits include balancing the healthcare allocation in city center and suburban area, avoiding overestimate the accessibility, and being much closer to reality, which have been referred by many pieces of literature [24,39,50]. In Scenario 2, we assumed that the attractiveness and travel time had equal influences on the tolerance function, but some of the results indicated that these two factors had different effects under various situations. Further analyses are suggested to be carried out for the MT2SFCA method with different weights of travel time and attractiveness. 

The maximum catchment areas of CHCs and hospitals used in this study were 30 min of walking and 30 min of driving. These values are decided according to the policy and studies focusing on the planning of healthcare facilities in China [22,33]. We also conducted a survey among local people in Shenzhen about ‘the farthest distance you can tolerate to visit CHCs/hospitals’. However, they were simplified values that do not consider the discrepancies among various population groups. Several studies found that the health equity usually varies among different groups, especially for the vulnerable groups [51,52]. Exploring the health equity of the multi-tiered medical system focusing on different population groups are recommended in further works. 

In this study, we considered two travel modes that aimed at two different healthcare types. The effects of travel modes on accessibility have been proposed by several studies and are debatable. In some studies, the demand or supply locations were scattered, and the different travel modes significantly affected the accessibility of the healthcare facility [53,54]. For some developed areas, the accessibility calculated for multi-travel modes resulted in similar results of those obtained for single travel mode [55]. However, various travel modes are still encouraged to be considered in further studies. 

It is also important to mention that the healthcare facilities used in this study were the facilities under the multi-tiered medical system, which included the private and public hospitals/CHCs. Private clinics were not considered. The reasons are that the private clinics cannot offer referral services, and the medical insurance is not available for most of them. As a result, the patients choosing the private clinics are mainly the floating population who are different from the long-term residents discussed in this study. However, the private clinics still help improve the accessibility and equity. Further work can focus on the effects of private clinics on current medical system and health equity.

There are other aspects that can be investigated in further works. Using the geographic center of administrative region to present population location is a traditional way when calculating accessibility and equity [20,23], whereas some studies proposed the effects of commute on routes when people visiting facilities [56,57]. It is recommended to consider the commuter-based locations in further work. The empirical validation of a certain function for a certain setting are regarded as an effective way to determine the appropriate distance decay function in traditional methods. Although the MT2SFCA method adapted the variety of the value distributions within the catchment area, the validation of the logistic cumulative distribution function used in MT2SFCA is still suggested by more empirical data.

## 7. Conclusions

In this study, we evaluated the accessibility and spatial equity of the multi-tiered medical system in China after the referral reform was introduced. Our study area was Shenzhen, China, and we proposed the MT2SFCA method, which was an improvement of the traditional M2SFCA method by replacing the distance decay function by the tolerance decay function using the formula of the logistic CDF. The CDF reshaped the function curve to adapt the distribution of tolerance values within the catchment area for the CHCs and hospitals in the multi-tiered medical system. When calculating the accessibility, we also considered the numbers of real outpatient visits and proposed two scenarios of tolerance to determine the effects of travel time and attractiveness when people select healthcare facilities, and then compared to the benchmark value. Travel time was set as the independent variable of decay function, which was referred to as the ‘benchmark’. In Scenario 1, we assumed that the tolerance was only affected by the attractiveness of facilities, and in Scenario 2, we assumed that the tolerance was influenced by both attractiveness and travel time. The outpatient visits normalized by the facility capacity represented the attractiveness of the facility.

By comparing the accessibility and spatial equity of the CHCs, hospitals, and the entire multi-tiered medical system, we deduced that the multi-tiered medical system could gain the advantages of both the CHCs and hospitals. The CHCs offset the lower accessibility of hospitals in suburban areas, and simultaneously, improved the accessibility to healthcare facilities. The hospitals balanced the inequity of accessibility caused by the CHCs. Thus, the selection of CHCs by patients relied more on travel time, whereas the facility features were more important when choosing hospitals. It was difficult to determine which factor affected the multi-tiered medical system more than the other. However, compared to the benchmark, both the scenarios portrayed lower accessibility in the central areas of the city, which indicated that the actual supply–demand relations of healthcare resources were not as optimistic as the results calculated using only the effects of travel time in the city center (via convenient transportation). Similarly, people in rural areas experienced slightly better accessibility to healthcare services, even though they had to travel longer distances.

The MT2SFCA method indicated remarkable results in evaluating the accessibility of the medical system. Under this two-way patient referral multi-tiered medical system, both the CHCs and hospitals were found to be crucial for the whole system. Only one type of healthcare facility could not reflect the real distribution of healthcare resources. Thus, when evaluating the accessibility and spatial equity of medical systems, various types of healthcare facilities must be considered. In further studies, factors other than travel time are recommended to be considered in the evaluation of healthcare equity and accessibility. Furthermore, commuter-based locations, various catchment sizes, different modes, population groups, and weights of contributing factors can be used to improve the MT2SFCA method. Further studies could move toward improving spatial accessibility and equity of healthcare services by building new facilities and pathways.

## Data Availability

Not applicable.
